# Discrimination of SARS-CoV-2 Infections From Other Viral Respiratory Infections by Scent Detection Dogs

**DOI:** 10.3389/fmed.2021.749588

**Published:** 2021-11-18

**Authors:** Nele Alexandra ten Hagen, Friederike Twele, Sebastian Meller, Paula Jendrny, Claudia Schulz, Maren von Köckritz-Blickwede, Ab Osterhaus, Hans Ebbers, Isabell Pink, Tobias Welte, Michael Peter Manns, Thomas Illig, Anahita Fathi, Marylyn Martina Addo, Andreas Nitsche, Andreas Puyskens, Janine Michel, Eva Krause, Rosina Ehmann, Albrecht von Brunn, Christiane Ernst, Katrin Zwirglmaier, Roman Wölfel, Alexandra Nau, Eva Philipp, Michael Engels, Esther Schalke, Holger Andreas Volk

**Affiliations:** ^1^Department of Small Animal Medicine and Surgery, University of Veterinary Medicine Hannover, Hannover, Germany; ^2^Research Center for Emerging Infections and Zoonoses, University of Veterinary Medicine Hannover, Hannover, Germany; ^3^Department of Biochemistry, University of Veterinary Medicine Hannover, Hannover, Germany; ^4^KynoScience Unternehmergesellschaft, Hörstel, Germany; ^5^Department of Respiratory Medicine, Hannover Medical School, Hannover, Germany; ^6^Hannover Medical School, Hannover, Germany; ^7^Hannover Unified Biobank, Hannover Medical School, Hannover, Germany; ^8^Department of Medicine, Division of Infectious Diseases, University Medical-Center Hamburg-Eppendorf, Hamburg, Germany; ^9^Department for Clinical Immunology of Infectious Diseases, Bernhard Nocht Institute for Tropical Medicine, Hamburg, Germany; ^10^German Center for Infection Research, Hamburg-Lübeck- Borstel-Riems, Hamburg, Germany; ^11^Center for Biological Threats and Special Pathogens (ZBS) 1, Highly Pathogenic Viruses, World Health Organisation Reference Laboratory for SARS-CoV-2 and World Health Organisation Collaborating Centre for Emerging Infections and Biological Threats, Robert Koch Institute, Berlin, Germany; ^12^Bundeswehr Institute of Microbiology, Munich, Germany; ^13^Max von Pettenkofer-Institute, Virology, Ludwig Maximilian University of Munich, Munich, Germany; ^14^German Center for Infection Research, Munich, Germany; ^15^Bundeswehr Medical Service Headquarters, Koblenz, Germany; ^16^Military Medical Center, Fürstenfeldbruck, Germany; ^17^Bundeswehr School of Dog Handling, Gräfin-Maltzan-Kaserne, Ulmen, Germany

**Keywords:** canine, volatile organic compound (VOC), COVID-19, screening test, coronavirus, SARS-CoV-2, scent detection dog

## Abstract

**Background:** Testing of possibly infected individuals remains cornerstone of containing the spread of SARS-CoV-2. Detection dogs could contribute to mass screening. Previous research demonstrated canines' ability to detect SARS-CoV-2-infections but has not investigated if dogs can differentiate between COVID-19 and other virus infections.

**Methods:** Twelve dogs were trained to detect SARS-CoV-2 positive samples. Three test scenarios were performed to evaluate their ability to discriminate SARS-CoV-2-infections from viral infections of a different aetiology. Naso- and oropharyngeal swab samples from individuals and samples from cell culture both infected with one of 15 viruses that may cause COVID-19-like symptoms were presented as distractors in a randomised, double-blind study. Dogs were either trained with SARS-CoV-2 positive saliva samples (test scenario I and II) or with supernatant from cell cultures (test scenario III).

**Results:** When using swab samples from individuals infected with viruses other than SARS-CoV-2 as distractors (test scenario I), dogs detected swab samples from SARS-CoV-2-infected individuals with a mean diagnostic sensitivity of 73.8% (95% CI: 66.0–81.7%) and a specificity of 95.1% (95% CI: 92.6–97.7%). In test scenario II and III cell culture supernatant from cells infected with SARS-CoV-2, cells infected with other coronaviruses and non-infected cells were presented. Dogs achieved mean diagnostic sensitivities of 61.2% (95% CI: 50.7–71.6%, test scenario II) and 75.8% (95% CI: 53.0–98.5%, test scenario III), respectively. The diagnostic specificities were 90.9% (95% CI: 87.3–94.6%, test scenario II) and 90.2% (95% CI: 81.1–99.4%, test scenario III), respectively.

**Conclusion:** In all three test scenarios the mean specificities were above 90% which indicates that dogs can distinguish SARS-CoV-2-infections from other viral infections. However, compared to earlier studies our scent dogs achieved lower diagnostic sensitivities. To deploy COVID-19 detection dogs as a reliable screening method it is therefore mandatory to include a variety of samples from different viral respiratory tract infections in dog training to ensure a successful discrimination process.

## Introduction

The ongoing coronavirus disease 2019 (COVID-19) pandemic caused by severe acute respiratory syndrome coronavirus 2 (SARS-CoV-2) still affects the life of people all over the world and highlights the need of rapid point-of-care screening tests as a key tool to contain viral spread. The real-time quantitative reverse transcription polymerase chain reaction (rtRT-PCR) is considered the gold standard for diagnosing SARS-CoV-2 infections with high diagnostic accuracy (1), but requires laboratory infrastructure, is time-consuming and can be cost-prohibitive and therefore of limited use for rapid field diagnosis during mass screening in public places, during large events or at ports-of-entry. In these situations, rapid point-of-care antigen tests are used for screening of individuals. However, a recently performed meta-analysis of rapid antigen test application indicates high variability of diagnostic accuracy under real-life conditions, with up to half of asymptomatic patients being tested false negative (2). Medical scent detection dogs could provide an additional screening tool. Several studies have proven canines' extraordinary olfactory acuity to detect individuals with infectious and non-infectious diseases (3). For example, they are capable of detecting a variety of cancer types like lung and breast cancer (4) malaria (5) and bacterial infections caused by *Clostridium difficile* (6), *Staphylococcus aureus* (7), and other bacteria (8). Consequently, several research groups currently train and deploy SARS-CoV-2 detection dogs, recently summarised in a WHO blueprint (9).

In the first published pilot study, dogs were able to detect saliva samples from COVID-19 patients with a diagnostic sensitivity of 83% and specificity of 96% (10), which has now been confirmed by multiple studies with larger sample sets and using the same or different body fluids (sweat or urine) (11–16). Interestingly, dogs are able to transfer their learned scent detection from beta-propiolactone (BPL) inactivated to non-inactivated samples as well as to different body fluids, with comparable diagnostic accuracies indicating a global, specific SARS-CoV-2-associated volatile compound release across different body secretions, independently from the patient being symptomatic or not (17). A recent interesting medical canine scent detection study has used mathematical modelling based on a large cohort of samples from symptomatic and asymptomatic SARS-CoV-2-infections with a wide range of virus loads, represented by varying cycle threshold (Ct) values, to show that these values had a negligible impact on sensitivity compared to lateral flow tests (18). In another recent publication, Hag-Ali and colleagues stated that scent dogs achieved even better sensitivities than the gold standard rtRT-PCR (19). Canine detection is also extremely rapid. Guest et al. report that just two dogs could screen 300 people in 30 min (18). All these studies demonstrate that scent detection dogs can discriminate between samples of SARS-CoV-2-infected, and non-infected healthy individuals with a high level of accuracy and speed. Detection dogs therefore may provide a reliable, fast (2–4 s per sample) screening method for SARS-CoV-2 infections, especially in countries with a lack of access to high-tech screening methods or as a preliminary mass screening for infectious diseases. However, until now, none of the studies evaluated if canines could also distinguish between SARS-CoV-2-infections and infections caused by different human coronaviruses nor other viruses that cause similar symptoms like influenza virus, parainfluenza virus or human rhinovirus. This has been criticised by reviewers (10). Thus, there is an urgent need to test COVID-19 medical scent detection dogs against other respiratory infectious diseases.

A pathogen-specific odour is thought to be detected by dogs being composed of unique volatile organic compounds (VOCs). Laboratory identification of the specific VOC pattern is, however, still in its infancy and there is little published data on the creation of different odours by viral infections. Angle et al. trained dogs to detect cell cultures infected with bovine viral diarrhoea virus (BVDV) (20). After training, these dogs were not just able to discriminate the BVDV-infected cell culture against an uninfected cell culture but also to cell cultures infected by bovine herpes virus 1 (BHV-1) and bovine parainfluenza virus 3 (BPIV-3) achieving high sensitivities and specificities (20). Aksenov and colleagues analysed VOCs emitted from cell cultures infected by different influenza virus subtypes and found unique VOC patterns for each subtype (21). A recent study has analysed breath samples from individuals infected with SARS-CoV-2 or influenza A virus and found also in this experiment virus-specific VOC patterns (22). These studies highlight that not only different virus families but also subtypes within one family have a different odour and could probably be distinguished by scent dogs. The aim of the current study was therefore to demonstrate that medical scent detection dogs can discriminate SARS-CoV-2 infections from other common viral respiratory tract infections, including other coronaviruses.

## Materials and Methods

Inactivated saliva samples from SARS-CoV-2-infected and healthy individuals as negative controls were used for training. The saliva samples were acquired and prepared as described in previous studies (10). In addition to the set-up of samples in our first study (10), which only included saliva and tracheobronchial secretion samples, the current study included diluted naso- and oropharyngeal swabs and supernatant from cell cultures infected with different respiratory viruses.

Clinical swab samples used in this study were obtained from routine diagnostics at the Robert Koch Institute (Berlin, Germany). For pooled SARS-CoV-2 samples, a mix of 20 naso- and oropharyngeal swabs from PCR confirmed SARS-CoV-2 patients were used, similar Ct values (<1.0 difference) were matched. A 1:3 dilution was performed using swab samples from healthy individuals in phosphate buffered saline (PBS) as a negative matrix. As sample medium, PBS was used for all samples. Furthermore, pooled samples from individuals infected with four different coronaviruses and nine other viruses causing respiratory infections in humans like influenza viruses and parainfluenza viruses were included (Table 1). All samples were tested negative via rtRT-PCR for all other viruses included. The status of each included sample was determined by rtRT-PCR (23, 24) at the Centre for Biological Threats and Special Pathogens, Highly Pathogenic Viruses (ZBS1), and Unit 17: Influenza and Other Respiratory Viruses, German National Influenza Centre RKI (Berlin, Germany).

**Table 1:** Viruses included in our studies.

**Virus**	**Swab sample**	**Cell culture sample**
Severe acute respiratory syndrome coronavirus type 2 (SARS-CoV-2)	X	X
Severe acute respiratory syndrome coronavirus type 1 (SARS-CoV-1)		X
Middle East respiratory syndrome-related coronavirus (MERS-CoV)		X
Human coronavirus 229E (HCoV-229E)	X	X
Human coronavirus OC43 (HCoV-OC43)	X	X
Human coronavirus NL63 (HCoV-NL63)	X	X
Human coronavirus HKU1 (HCoV-HKU1)	X	
Influenza A virus subtype H1N1 (A/H1N1)	X	
Influenza A virus subtype H3N2 (A/H3N2)	X	
Influenza B virus subtype Yamagata (B/YAM)	X	
Human respiratory syncytial virus (RSV)	X	
Human metapneumovirus (HMPV)	X	
Human parainfluenza virus type 1 (HPIV-1)	X	
Human parainfluenza virus type 3 (HPIV-3)	X	
Rhinovirus	X	
Adenovirus	X	

Samples originating from cell culture were derived from the Bundeswehr Institute of Microbiology (Munich, Germany). Six different human coronaviruses were cultured in a human hepatocyte derived carcinoma cell line (HuH7.5 cells, see Table 1). Subconfluent to confluent monolayers of HuH7.5 cells were rinsed with serum-free medium and inoculated with SARS-CoV-1 strain “Frankfurt-1” (kindly provided by C. Drosten, Charité Berlin), SARS-CoV-2 strain “BavPat1/2020,” (Global Initiative on Sharing All Influenza Data acc. no. EPI_ISL_406862), Middle East respiratory syndrome-related coronavirus (MERS-CoV) strain “EMC” (kindly provided by Bart Haagmans, Erasmus Medical Center Rotterdam), human coronavirus 229E (HCoV-229E) (American Type Culture Collection (ATCC) acc. no. VR-740), HCoV-OC43 (ATCC acc. no. VR-1558) and HCoVNL63. A mock control was included and handled identically with serum-free medium as the inoculum. After incubation for 60 min, the inoculum was discarded, the monolayers were rinsed three times with serum-free medium before supplementing the cells with minimum essential medium (MEM) containing 2% foetal calf serum. Incubation was performed at 33°C, 5% CO_2_ and 90% humidity for HCoV-OC43 and -NL63 and at 37°C for the other coronaviruses. Supernatant of the cell cultures was harvested when infection of the monolayers reached 90–100% [assessed either by cytopathic effect or immunofluorescence signal against viral protein (data not shown)]. Supernatants were cleared from cellular debris by centrifugation at 5,000 xg for 10 min in an Eppendorf 5804 centrifuge. Cleared supernatants were inactivated as described in our first study (10). Inactivation success was assessed by lack of growth on Vero E6 cells for SARS-CoV-1, SARS-CoV-2, and MERS-CoV and inoculation of HuH7.5 cells for HCoV-229E, -NL63, and -OC43. Virus identity and culture success were further confirmed by quantitative RT-PCR (25). Inactivated supernatants were aliquoted and stored at −20°C for storage and at 4°C for subsequent use (Supplementary Table 1).

Based on former results showing that BPL inactivation does not change scent dog detection (17) and for easier and safer handling of samples, all samples for either training and testing were BPL inactivated as formerly described (10). Until usage, the samples were deep-frozen at −80°C. For training and testing, a volume of 100 μl per sample was pipetted onto a cotton swab which was placed into a 4 ml glass tube. All samples were handled by the same two persons wearing disposable gloves to prevent odour contamination.

In total, twelve dogs (four males and eight females) were included in our studies. All dogs completed obedience training before the study, and some had a history of scent detection or protection work. Ages ranged between 1 and 5 years. Included dog breeds were Labrador Retriever (*n* = 5), Malinois (*n* = 4), German Shepherd (*n* = 2) and Cocker Spaniel (*n* = 1) (Supplementary Table 2).

As described in our previous studies (10, 17) a device called “Detection Dog Training System” (DDTS, Kynoscience UG, Hörstel, Germany) was used for sample presentation and positive reinforcement during training and testing. The DDTS allows for rapid, automatic, randomised, trainer-bias devoid and double-blind sample presentation (10). To verify the recorded results of the DDTS the dogs were filmed during testing and the videos were analysed manually. The training method is based on classical and operant conditioning by using only positive reinforcement as previously described in Jendrny et al. (10, 17). In the present study, the training period lasted 3 days with a high number of sample presentations using inactivated positive saliva samples, or the supernatants of cell cultures infected by SARS-CoV-2 as positive samples. As control samples, negative saliva from healthy individuals (SARS-CoV-2 rtRT-PCR negative) or the supernatant of a non-infected cell culture were utilised. Apart of the “green” dogs used in scenario III, all dogs completed previous training in 2020 for detection of saliva samples of SARS-CoV-2 infected individuals. They were still able to distinguish positive and negative samples even though they had not been trained with SARS-Cov-2 samples for 5 months. After training, the double-blind study was conducted on 2 days using cell cultures and pooled swab samples. In the test scenario I, SARS-CoV-2 positive naso- and oropharyngeal pooled swab samples were utilised as target odours. Distractors were swab samples from patients infected by other viruses causing respiratory tract infections including different coronaviruses (Table 1). In the following experiments (test scenario II and III) supernatant from cell cultures infected by several coronaviruses including SARS-CoV-2 and non-infected cell cultures was presented to evaluate if medical scent detection dogs could discriminate between SARS-CoV-2 infection and infection with other coronaviruses or negative controls.

Every nose dip into the DDTS' slots was evaluated with four possible options:

1. True positive (TP): the dog correctly indicates a SARS-CoV-2 positive sample
2. False negative (FN): the dog sniffs shortly at a SARS-CoV-2 positive sample but does not indicate it
3. True negative (TN): the dog sniffs shortly at a negative/distractor sample and correctly does not indicate it
4. False positive (FP): the dog incorrectly indicates a negative/distractor sample

For indicating a sample, the dogs rested with their snout in the respective device test slot (“freezing”). The indication time was recorded by the DDTS and after indicating the target sample the device automatically responded with a reward, i.e., food. Afterwards, the DDTS changed the positions of the presented samples without letting the dog or dog handler know the new positions of negative, distractor or positive samples. This allowed a double-blind sample presentation. In addition, all staff involved was positioned to prevent any interaction or influencing of the animals during the study.

The diagnostic sensitivity as well as diagnostic specificity, positive predictive values (PPV), and negative predictive values (NPV) were calculated according to Trevethan (26). PPV is defined as the probability that people with a positive screening test result indeed do have the condition of interest and was calculated as [true positive/(true positive + false positive)] ×100. NPV is defined as the probability that people with a negative screening test result do not have the condition of interest and was calculated [true negative/(false negative + true negative)] ×100. 95% confidence intervals (CIs) for sensitivity, specificity, PPV, and NPV were calculated with the hybrid Wilson/Brown method (27). Means of sensitivity, specificity, PPV, NPV, and accuracy with corresponding 95% CIs were also calculated per scenario. Two-tailed Fisher's exact test was used for analysis of the contingency tables; a *P* ≤ 0.05 was considered significant. All calculations were done with the Prism 9 software from GraphPad (La Jolla, CA, USA).

This study was carried out in accordance with the ethical requirements established by the Declaration of Helsinki. The study obtained ethical approval by the Berlin Chamber of Physicians (Eth 20/40) and was approved by the local Ethics Committee of Hannover Medical School (MHH) and Hamburg Medical Association for the University Medical-Center Hamburg-Eppendorf (UKE) (ethic consent number 9042_BO_K_2020 and PV7298, respectively). Written informed consent from all participants was obtained before sample collection. Animal work according to the study protocol and design was approved by the German Armed Forces.

## Results

**Figure 1:**
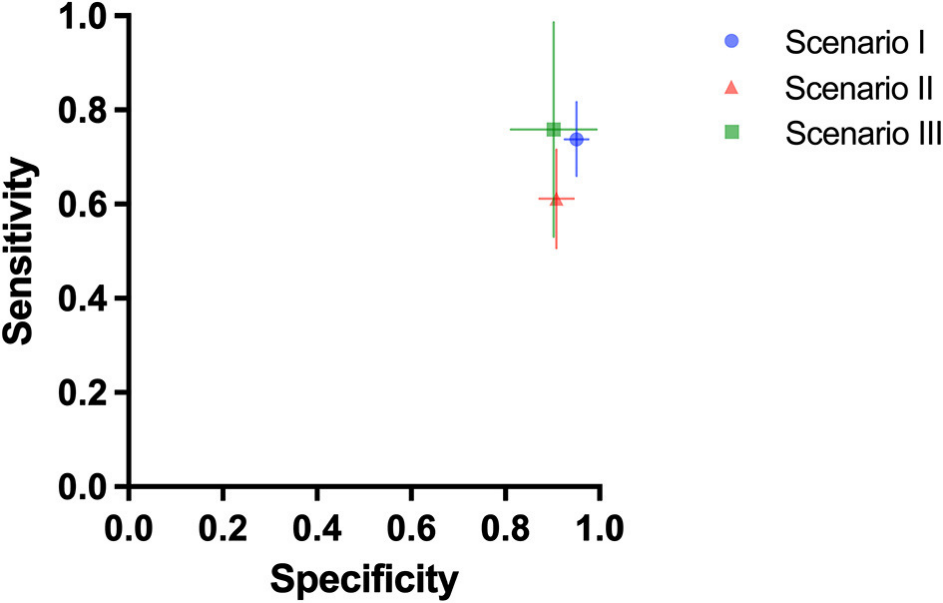
Mean diagnostic specificity and sensitivity for all dogs for swab samples with preceding saliva training (test scenario I: blue circle), cell culture samples with preceding saliva training (test scenario II: red triangle), and cell culture samples with preceding cell culture training (test scenario III: green square), respectively. The 95% confidence intervals for specificity and sensitivity are shown with horizontal and vertical lines, respectively.

Three test scenarios were performed to address the aim of the study. In the first test scenario (scenario I), dogs who were trained with saliva samples discriminated between SARS-CoV-2 swab samples and swab samples from patients infected by other respiratory tract infection viruses (Table 1). The dogs achieved a mean sensitivity of 73.8% (95% CI: 66.0–81.7%) and a specificity of 95.1% (95% CI: 92.6–97.7%). In the following test scenario (scenario II) cell culture supernatants from cells infected by different coronaviruses or non-infected cells were comparatively presented to the dogs. Dogs were able to discriminate the SARS-CoV-2 supernatant from supernatants from other coronaviruses and non-infected controls with a mean sensitivity of 61.2% (95% CI: 50.7–71.6%) and a specificity of 90.9% (95% CI: 87.3–94.6%). In the last test scenario (scenario III), not formerly trained (“green”) dogs were directly trained using the supernatant from cell cultures infected by SARS-CoV-2. The dogs achieved a mean sensitivity of 75.8% (95% CI: 53.0–98.5%) and a specificity of 90.2% (95% CI: 81.1–99.4%) (Table 2; Figure 1).

**Table 2:** Diagnostic performance of the scent detection dogs.

**Test scenario**	**Dog**	**Detection**	**SARS-CoV-2 infection status**		**Total number of sample presentations**	**Diagnostic specificity (Sp)**	**Diagnostic sensitivity (Se)**	**Confidence interval (95% CI) Sp**	**Confidence interval (95% CI) Se**	**Positive predictive value (PPV)**	**Negative predictive value (NPV)**	**Confidence interval (95% CI) PPV**	**Confidence interval (95% CI) NPV**	**Accuracy**	**Fisher's exact test *p*-value**
			**Positive**	**Negative/other pathogen**											
I) Swab samples (after	Dog 1	Yes	16	8	132	0.926	0.667	0.861–0.962	0.467–0.82	0.667	0.926	0.467–0.82	0.861–0.962	0.879	<0.0001
training with inactivated		No	8	100											
saliva samples)	Dog 2	Yes	19	6	154	0.954	0.826	0.904–0.979	0.629–0.93	0.76	0.969	0.566–0.886	0.923–0.988	0.935	<0.0001
		No	4	125											
	Dog 3	Yes	16	6	139	0.948	0.667	0.891–0.976	0.467–0.82	0.727	0.932	0.518–0.868	0.871–0.965	0.899	<0.0001
		No	8	109											
	Dog 4	Yes	17	12	143	0.899	0.708	0.832–0.941	0.508–0.851	0.586	0.939	0.407–0.745	0.879–0.97	0.867	<0.0001
		No	7	107											
	Dog 5	Yes	18	7	154	0.946	0.75	0.893–0.974	0.551–0.88	0.72	0.953	0.524–0.857	0.902–0.979	0.916	<0.0001
		No	6	123											
	Dog 6	Yes	15	0	164	1	0.6	0.973–1	0.407–0.766	1	0.933	0.796–1	0.881–0.963	0.939	<0.0001
		No	10	139											
	Dog 7	Yes	18	4	122	0.96	0.818	0.902–0.984	0.615–0.927	0.818	0.96	0.615–0.927	0.902–0.984	0.934	<0.0001
		No	4	96											
	Dog 8	Yes	20	3	146	0.976	0.87	0.931–0.993	0.679–0.955	0.87	0.976	0.679–0.955	0.931–0.993	0.959	<0.0001
		No	3	120											
						**Mean Sp**	**Mean Se**	**95% CI of mean Sp**	**95% CI of mean Se**	**Mean PPV**	**Mean NPV**	**95% CI of mean PPV**	**95% CI of mean NPV**	**Mean accuracy**	**95% CI of mean accuracy**
						0.951	0.738	0.926–0.977	0.66–0.817	0.768	0.948	0.662–0.875	0.933–0.964	0.916	0.889–0.943
II) Cell culture samples	Dog 1	Yes	8	6	75	0.905	0.667	0.807–0.956	0.391–0.862	0.571	0.934	0.326–0.786	0.843–0.974	0.867	<0.0001
(after training with		No	4	57											
inactivated saliva	Dog 2	Yes	4	3	53	0.933	0.5	0.821–0.977	0.215–0.785	0.571	0.913	0.25–0.842	0.797–0.966	0.868	0.0068
samples)		No	4	42											
	Dog 3	Yes	7	7	65	0.865	0.538	0.747–0.933	0.291–0.768	0.5	0.882	0.268–0.732	0.766–0.945	0.8	0.0042
		No	6	45											
	Dog 4	Yes	8	2	79	0.97	0.615	0.896–0.995	0.355–0.823	0.8	0.928	0.49–0.964	0.841–0.969	0.911	<0.0001
		No	5	64											
	Dog 5	Yes	9	2	45	0.941	0.818	0.809–0.99	0.523–0.968	0.818	0.941	0.523–0.968	0.809–0.99	0.911	<0.0001
		No	2	32											
	Dog 6	Yes	8	11	75	0.82	0.571	0.705–0.896	0.326–0.786	0.421	0.893	0.231–0.637	0.785–0.95	0.773	0.0051
		No	6	50											
	Dog 7	Yes	7	2	51	0.946	0.5	0.823–0.99	0.268–0.732	0.778	0.833	0.453–0.961	0.694–0.917	0.824	0.0008
		No	7	35											
	Dog 8	Yes	10	5	81	0.928	833	0.841–0.969	0.552–97	0.667	0.97	0.417–0.848	0.896–0.955	0.914	<0.0001
		No	2	64											
	Dog 9	Yes	6	7	69	0.875	0.462	0.764–0.938	0.232–0.709	0.462	0.875	0.232–0.709	0.764–0.938	0.797	0.0119
		No	7	49											
						0.909	0.621	0.873–0.946	0.507–0.716	0.621	0.908	0.505–0.737	0.876–0.939	0.852	0.81–0.894
						**Mean Sp**	**Mean Se**	**95% CI of mean Sp**	**95% CI of mean Se**	**Mean PPV**	**Mean NPV**	**95% CI of mean PPV**	**95% CI of mean NPV**	**Mean accuracy**	**95% CI of mean accuracy**
						0.951	0.738	0.926–0.977	0.66–0.817	0.768	0.948	0.662–0.875	0.933–0.964	0.916	0.889–0.943
III) Cell culture samples	Dog 1	Yes	10	6	44	0.818	0.909	0.656–0.914	0.623–0.995	0.625	0.964	0.386–0.815	0.823–0.998	0.841	<0.0001
(after training with cell		No	1	27											
culture samples)	Dog 2	Yes	10	0	30	1	1	0.839–1	0.722–1	1	1	0.722–1	0.839–1	1	<0.0001
		No	0	20											
	Dog 3	Yes	7	10	78	0.851	0.636	0.747–0.917	0.354–0.848	0.412	0.934	0.216–0.64	0.843–0.974	0.821	0.0014
		No	4	57											
	Dog 4	Yes	9	3	76	0.951	0.6	0.865–0.987	0.357–0.802	0.75	0.906	0.468–0.911	0.81–0.956	0.882	<0.0001
		No	6	58											
	Dog 5	Yes	9	7	79	0.892	0.643	0.794–0.947	0.388–0.837	0.563	0.921	0.332–0.769	0.827–0.966	0.848	<0.0001
		No	5	58											
						**Mean Sp**	**Mean Se**	**95% CI of mean Sp**	**95% CI of mean Se**	**Mean PPV**	**Mean NPV**	**95% CI of mean PPV**	**95% CI of mean NPV**	**Mean accuracy**	**95% CI of mean accuracy**
						0.902	0.758	0.811–0.994	0.53–0.985	0.67	0.945	0.396–0.944	0.899–0.991	0.878	0.79–0.967

**Figure 2:**
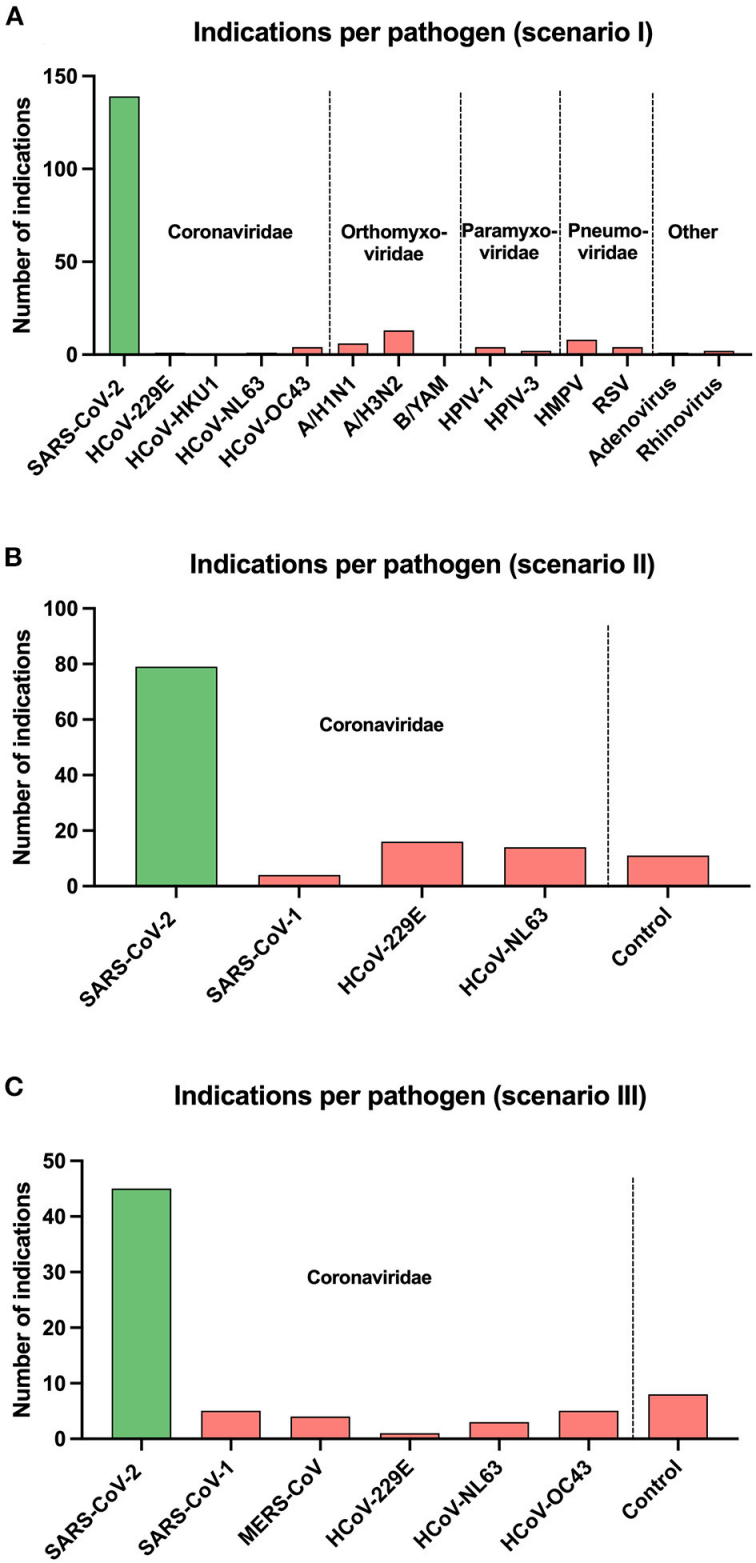
Overall number of indications per pathogen for each test scenario of scent dogs is shown as vertical bars: **(A)** test scenario I (swab samples with preceding saliva training), **(B)** test scenario II (cell culture samples with preceding saliva training), and **(C)** test scenario III (cell culture samples with preceding cell culture training). Pathogens are grouped on the x-axis according to their virus families and separated by vertical dotted lines. *Coronaviridae*: severe acute respiratory syndrome coronavirus 1 and 2 (SARS-CoV-1 and SARS-CoV-2), Middle East respiratory syndrome-related coronavirus (MERS-CoV), human coronavirus 229E (HCoV-229E), human coronavirus HKU1 (HCoV-HKU1), human coronavirus NL63 (HCoV-NL63), human coronavirus OC43 (HCoV-OC43). *Orthomyxoviridae*: influenza A virus subtype H1N1 (A/H1N1), influenza A virus subtype H3N2 (A/H3N2), influenza B virus subtype Yamagata (B/YAM). *Paramyxoviridae*: human parainfluenza virus type 1 (HPIV-1), human parainfluenza virus type 3 (HPIV-3). *Pneumoviridae*: human metapneumovirus (HMPV), respiratory syncytial virus (RSV).

Overall, a total of 2,054 sample presentations were performed in three different test scenarios. During the presentation of swab samples (test scenario I), 139 correct indications and 50 false rejections of SARS-CoV-2 positive swab samples were recorded, while 919 correct rejections and only 46 false indications of swab samples from the other 13 viruses were made. When the supernatants of the different cell cultures were presented to the dogs trained with saliva (test scenario II), they indicated 67 SARS-CoV-2 sample presentations correctly and rejected 43 positive samples incorrectly. They made 45 false positive responses to any one of three other coronaviruses or one non-infected control and 443 correct rejections. In comparison, dogs trained with cell cultures for 3 days (test scenario III) correctly identified 45 SARS-CoV-2 supernatants correctly but incorrectly rejected 16 positive samples, whereas 220 correctly negative and 26 false positive responses to the other seven cell culture samples (six different coronaviruses and one non-infected control) were made (Figure 2).

## Discussion

Rapid detection of SARS-CoV-2 infections remains one of the main strategies to control the current global pandemic. Several studies have shown that trained scent detection dogs can discriminate SARS-CoV-2-infected individuals from healthy individuals with high diagnostic accuracies. However, to use medical detection dogs, as a reliable diagnostic test, it is important to ensure their capability to distinguish between samples from SARS-CoV-2-infected people and other viral infections causing similar symptoms. In the current study, dogs were able to discriminate samples from SARS-CoV-2 infected individuals and cell cultures from those infected with one of 15 other common acute respiratory viruses as distractors.

During 964 presentations of swab samples from patients with different viral infections other than SARS-CoV-2 (test scenario I), the scent dogs alerted falsely to 46 samples, which led to a mean specificity of 95.1%. This specificity is comparable to our first pilot study by Jendrny et al. in which we only used samples from healthy, SARS-CoV-2 negative individuals without any respiratory symptoms as control samples and obtained a specificity of 96.35% (10). When using cell culture samples (test scenario II and III), the canines achieved mean specificities of 90.2% and 90.6%, respectively. Although the diagnostic specificities were found lower compared to our previous studies (10, 17), our results indicate that dogs can distinguish SARS-CoV-2 from other viral respiratory infections.

When presenting cell culture samples to dogs trained by saliva, the mean sensitivity was 61.2%, which is lower compared to sensitivities ranging from 82% to 95% in our previous studies (10, 17). The reduced performance in some dogs could be explained by not entirely identical VOC pattern that is released from cell culture supernatant compared to VOCs originating from saliva samples of the organism, the human body. In contrast to this observation, some of the dogs were able to directly transfer from saliva samples to supernatant samples. Possible explanations for this inconsistency might be that not every dog is conditioned to exactly the same VOC pattern or that individual dogs may not recognise identical VOC patterns. It is unknown which disease-specific VOCs are detected by the dogs and it is reasonable that the dogs learned slightly different VOC patterns as positive. Despite this, results from previous studies demonstrate that all dogs could be successfully trained to a disease-specific odour (10, 17). It would have been best if we had used saliva samples from individuals with different viral infections for testing. Unfortunately, this was not possible, as there were currently few infections with other respiratory viruses due to the high hygiene standards established during SARS-CoV-2 pandemic to prevent virus transmission and spread. Cell cultures provide the opportunity to generate odour samples independent of the availability of acute patients and it is possible to include samples of a wide range of different viruses, including other coronaviruses. Therefore, we decided to directly train five dogs with cell culture supernatant from human cells infected by SARS-CoV-2 instead of saliva and assess their diagnostic performance in a subsequent session. After just 3 days of training the mean sensitivity increased from 61.2% for the dogs trained with saliva samples to 75.8% for the five dogs trained with SARS-CoV-2 cell culture supernatant. A longer training period likely would have increased diagnostic accuracies (20).

The prevalence in our study was 17.5%. The high prevalence is a result of the fact that we presented one SARS-CoV-2 positive sample next to several samples from different viruses. In a pandemic, prevalences will vary and are usually significantly lower. When the prevalence falls below a certain threshold the dogs might get frustrated and stop searching for a positive sample. To ensure that scent dogs keep their diagnostic accuracies in case of a low prevalence and to have an internal control it is important to reward dogs during a test run not only for finding positive samples but also for not indicating wrongly negative samples. Furthermore, one has to ensure that the training entails an increasing number of empty runs (presentation of only negative samples). Dogs' frustrations levels should then be recorded and considered when working in the field, ensuring dogs will receive a sufficient number of rewards to keep them positively engaged in the field. A further limitation can be the dog handler. Not only the dog but also the dog's handler has an individual character, an individual training level and therefore require different training requirements. The DDTS can overcome some of these limitations, as dogs can be trained to work independently [Supplementary Video 1 and see Jendrny et al. (10)].

The prevalence of an infectious diseases is dynamic in a pandemic. As aforementioned the prevalence in our test paradigm was higher than in the current pandemic situation, which is further subject to change with an ever-increasing number of people getting vaccinated. The real positive predictive values would be with the current prevalence lower when sensitivity and specificity of dogs remain unchanged and the dogs would be deployed without a rewarding system. However, as in our test setting, a lower prevalence does not impact the performance of the dogs necessarily, as the frequency of getting rewarded is above the prevalence of the disease.

The disease-specific odour that can be detected by dogs is thought to be determined by a specific pattern of VOCs. VOCs are produced by cell metabolism and released with breath, urine, saliva, blood, faeces, sweat and other body fluids (28). In comparison to bacteria, viruses have no own metabolism, but the common hypothesis is that viruses can change the metabolism of the infected host cell and therefore determine a special VOC pattern (29). The composition of emitted VOCs in human body fluids are not only a result of non-infectious and infectious diseases but depend on a variety of factors such as age, sex, and diet (30). Every human, regardless of an infection, emits a variety of VOCs in a special pattern, which is called the human volatilome, and this pattern determines the unique body odour (28, 31). The usage of cell cultures provides the opportunity to exclude a lot of these influencing factors. Human samples contain a wide array of virus-independent VOCs and dogs need to seek out the disease-specific odour. When training with samples from individuals it is necessary to include a large number of human subjects to ensure that dogs are conditioned on the disease-specific odour. Training with SARS-CoV-2-infected cell cultures as the target odour and an equally treated, non-infected cell culture as negative control sample possibly simplifies the discrimination process for the dog between infected and non-infected samples. For our studies HuH7.5, a well differentiated cell line, was utilised to produce samples from different coronavirus infections and a non-infected cell culture as control. HuH7.5 cells were originally derived from a liver tumour in a 57-year-old Japanese male in 1982. HuH7.5 are particularly used for propagating the hepatitis C virus *in vitro* (32) and provided the opportunity to cultivate six different coronaviruses in the same human cellular background. For all cell culture samples the same serum-free medium and MEM containing 2% foetal calf serum were used to prevent odour interferences. Therefore, the odours of the cell culture samples were not affected by individual factors of the host like age, sex, diet or underlying medical conditions which are usually influencing odour samples. Consequently, the differences in the emitted VOCs between the infected cell culture samples are based on the specific coronavirus. However, cell culture samples do not take into account that during the infection a lot of changes occur in the infected organs and organism, like the complex mix of inflammatory reaction and cellular influx with specific se- and excretion and debris of dying cells. Obviously, this is not mimicked in a cell culture and may at least in part explain some of the discrepancies between swab sample and cell culture results. Consequently, when training for real-life deployment samples from infected individuals should be preferred. However, regardless the complex changes in VOC profile in infected individuals our cell culture results indicate that the VOCs created by infected cells are virus specific, which is why scent dogs can discriminate cells infected with different viruses. Furthermore, apart from scenting VOC patterns, dogs might also be capable of detecting directly viruses or viral proteins via their vomeronasal organ. The vomeronasal organ is capable of processing a wide variety of molecules, including proteins, thus representing a different and additional mechanism of odour perception (33, 34), which could explain dogs being able to discriminate specific viruses.

Several studies have evaluated the VOCs of viral infections (21, 35–37). It has been shown, that human tracheobronchial epithelial (TBE) cells infected with human rhinovirus (HRV) emit distinct VOCs compared to non-infected cells and cells inoculated with inactivated HRV (38). In a follow-up study, TBE cells were infected with HRV or influenza A virus subtype H1N1 (A/H1N1) with corresponding non-infected controls. Emitted VOCs were analysed via gas chromatography-mass spectrometry. Fifty-four unique VOCs were found distinguishing virus-infected from -uninfected cells. Forty of these VOCs were specific for A/H1N1-infected cells, but five occurred in A/H1N1- and HRV-VOC patterns (35). In addition, infections by different influenza A virus subtypes result in disparate VOC patterns (21). Current data from several studies suggest that SARS-CoV-2 infections create a specific VOC pattern which could be used in diagnostics (22, 37, 39, 40). Steppert et al. analysed exhaled breath from persons infected by SARS-CoV-2 or influenza A virus and healthy people via multi-capillary column-ion mobility spectrometry. They were able to discriminate between SARS-CoV-2, influenza A virus and controls in a few minutes which indicates that SARS-CoV-2 and influenza A virus infections can be distinguished by their differing VOC patterns (22). In summary, these data indicate that every viral infection creates its own specific VOC pattern and can therefore be discriminated.

Preliminary work on discriminating different viral infections by scent dogs was performed by Angle et al. (20). They used cell cultures infected with BVDV as the target sample and cell cultures infected with BHV-1 or BPIV-3 as distractors presented in a scent wheel with eight arms. After a training period of 2 months, the two dogs achieved sensitivities of 85% and 96.7% and specificities of 98.1% and 99.3% (20). These results indicate that trained dogs can discriminate different viral infections by their odour which is in accordance with our findings. A successful discrimination is fundamental in scent detection training, meaning to be able to differentiate the target odour from similar odours (41). The main aim of our study was to prove that dogs can discriminate different viral respiratory tract infections by their odour. In all three test scenarios our dogs achieved mean specificities above 90% which indicates their capability to distinguish SARS-CoV-2 infections from infections with other viruses. However, in contrast to earlier findings our scent dogs achieved lower diagnostic accuracies (10, 17). This discrepancy could be attributed to a more similar odour of SARS-CoV-2 infections to other viral infections than to non-infected individuals or cells. The similar odour of different viral infections probably resulted in a lack of discrimination and should be considered in subsequent training and testing. Our study clearly shows that it is mandatory to include other viruses in dog training to keep the diagnostic accuracy high. Our results indicate that presenting samples from different viral infections in the early training phase would improve the dogs' diagnostic skills and will support a successful discrimination process. This would ensure that scent dogs are conditioned to the unique smell of a SARS-CoV-2 infection and not to additional VOCs which are produced by several viral infections.

## Conclusion

In the current situation rapid antigen tests are used for screening people for SARS-CoV-2 infections, which generate test results within 15 min. Manufacturers state sensitivities above 90% (42), but several studies determined significantly lower sensitivities with certain tests (2, 43). The WHO and the Paul Ehrlich Institute (PEI, Langen, Germany) recommend a sensitivity of ≥80% and a specificity of ≥97% for rapid antigen tests (44, 45). In real life settings while screening asymptomatic people, Dinnes et al. found a mean sensitivity of 58% (2). For their deployment as a reliable diagnostic test COVID-19 detection dogs should meet the criteria recommended by the WHO and national institution like PEI. Previous studies indicate that the scent dog method could meet these criteria (14, 17, 19). In several studies dogs showed their capability to distinguish SARS-CoV-2 positive samples from negative samples with high diagnostic accuracy regardless of training method or sample type (11, 12, 14–19). Our results demonstrate their ability to differentiate viral respiratory tract infections by their odour but suggest including a variety of viruses during dog training to guarantee a high diagnostic accuracy. Further research should be performed to validate dogs' scent recognition capabilities as diagnostic tool, especially in asymptomatic or pre-symptomatic patients, vaccinated or not, as infected individuals spread virus and could even be super-spreaders, as has been documented (46). Follow-up study in this category is needed by testing larger cohort of swabs from asymptomatic rtRT-PCR positive individuals.

## Data Availability Statement

The original contributions presented in the study are included in the article/Supplementary Material, further inquiries can be directed to the corresponding author.

## Ethics Statement

The studies involving human participants were reviewed and approved by Berlin Chamber of Physicians (Eth 20/40) Hannover Medical School 9042_BO_K_2020 University Medical-Center Hamburg-Eppendorf PV7298. The patients/participants provided their written informed consent to participate in this study. The animal study was reviewed and approved by German Armed Forces.

## Author Contributions

NH participated in the planning of the study, carried out the main practical work, data analyses, and helped draught the manuscript. FT, SM, PJ, and HV designed and coordinated the study, drafted the manuscript (FT), conducted and coordinated (FT) the sample acquisition, and were responsible for data analyses (SM). MK-B, CS, and AO participated in the planning of the laboratory part of the study and were in charge for the legal permission for sample processing. CS, RE, KZ, and RW carried out the laboratory work at the Research Center for Emerging Infections and Zoonoses (CS) and Bundeswehr Institute of Microbiology (RE, KZ, and RW) including sample preparation, virus inactivation, and RtRT-PCR. HE programmed the DDTS software and supported the dog training. IP, TW, MM, AF, and MA were in charge for the ethical approval, patient recruitment and sample collection (IP and AF) at Hannover Medical School (IP, TW, and MM), and University Medical-Center Hamburg-Eppendorf (AF and MA). ANi and AP coordinated the sample acquisition at the Robert Koch Institute. AP, JM, and EK performed the sample preparation including rtRT-PCR at the Robert Koch Institute. ANa and EP were responsible for coordinating the acquisition and data of samples as part of the support provided by the German armed forces. CE was responsible for the special research proposal of the German Armed Forces as project manager on the part of the German Armed Forces. ME coordinated the cooperation with the University of Veterinary Medicine Hannover. AB participated in the planning of the cultivation of the different coronaviruses and provided the HuH7.5 cells. ES was responsible for the dog training and helped with data analyses. ME and ES were also involved in designing and coordinating the study. All authors have read and approved the final manuscript.

## Funding

The project was funded as a special research project of the German Armed Forces Medical Service (02Z9-S-852121) and supported by the COVID-19 Research Network of the State of Lower Saxony (COFONI) through funding from the Ministry of Science and Culture of Lower Saxony in Germany (14-76403-184).

## Conflict of Interest

The authors declare that the research was conducted in the absence of any commercial or financial relationships that could be construed as a potential conflict of interest. The handling editor is currently organizing a Research Topic with one of the authors HV.

## Publisher's Note

All claims expressed in this article are solely those of the authors and do not necessarily represent those of their affiliated organizations, or those of the publisher, the editors and the reviewers. Any product that may be evaluated in this article, or claim that may be made by its manufacturer, is not guaranteed or endorsed by the publisher.

## Supplementary Material

Supplementary Table 1 Characteristics of the samples used for the study.

Supplementary Video 1 Scent detection dog working with Detection Dog Training System (DDTS). The video shows the Malinois “Filou” during a detection session. At each detection run only one hole is presenting the target scent, in this case SARS-CoV-2 samples, with the other six holes presenting the distractors. The dog indicates a positive detection by remaining with the nose longer than 2 s in the hole. This will release a food reward, a beeping sound and will be recorded automatically. The device then rearranges the sample presentation automatically and randomly.
