# Supplementary material for: Discrimination of SARS-CoV-2 Infections From Other Viral Respiratory Infections by Scent Detection Dogs
Source: Front Med (Lausanne). 2021 Nov 18;8:749588. doi: 10.3389/fmed.2021.749588 (PMC8636992; doi:10.3389/fmed.2021.749588)
Supplement: Supplementary file 3 [file Table_2.DOCX]

| **Name** | **Sex** | **Age (years)** | **Breed** | **Specialty** |
| --- | --- | --- | --- | --- |
| Lotta | female | 5 | Labrador Retriever | Explosive detection dog |
| Filou | female | 3 | Malinois | Mine detection dog |
| Füge | female, castrated | 4 | German Shepherd | No previous training except obedience |
| Vine | female, castrated | 5 | Malinois | No previous training except obedience |
| Bellatrix | female | 1 | Labrador Retriever | No previous training except obedience |
| Margo | female | 1 | Labrador Retriever | No previous training except obedience |
| Erec junior | male | 3 | Malinois | Explosive detection and protection work |
| Billy | male | 1,5 | Labrador | No previous training |
| Harry | male | 1,5 | Labrador | No previous training |
| Peggy | female | 1 | Malinois | No previous training |
| Baila | female | 3,5 | German Shepherd | Tracking, Obedience,  Protection work |
| Joe | male | 1,5 | Cocker Spaniel | Blood detection |
